# Live imaging of the airway epithelium reveals that mucociliary clearance modulates SARS-CoV-2 spread

**DOI:** 10.21203/rs.3.rs-3246773/v1

**Published:** 2023-09-08

**Authors:** Thomas Hope, Mark Becker, Laura Martin-Sancho, Lacy Simons, Michael McRaven, Sumit Chanda, Judd Hultquist

**Affiliations:** Northwestern University; Northwestern University; Imperial College London; Northwestern University; Northwestern University; Sanford Burnham Prebys Medical Discovery Institute; Northwestern University

**Keywords:** epithelium, airway, sars-cov-2, microscopy, live cell imaging, cilia, mucus, mucociliary clearance, innate immunity, respiratory virus, airway epithelial cells

## Abstract

SARS-CoV-2 initiates infection in the conducting airways, which rely on mucocilliary clearance (MCC) to minimize pathogen penetration. However, it is unclear how MCC impacts SARS-CoV-2 spread after infection is established. To understand viral spread at this site, we performed live imaging of SARS-CoV-2 infected differentiated primary human bronchial epithelium cultures for up to 9 days. Fluorescent markers for cilia and mucus allowed longitudinal monitoring of MCC, ciliary motion, and infection. The number of infected cells peaked at 4 days post-infection in characteristic foci that followed mucus movement. Inhibition of MCC using physical and genetic perturbations limited foci. Later in infection, MCC was diminished despite relatively subtle ciliary function defects. Resumption of MCC and infection spread after mucus removal suggests that mucus secretion mediates this effect. We show that MCC facilitates SARS-CoV-2 spread early in infection while later decreases in MCC inhibit spread, suggesting a complex interplay between SARS-CoV-2 and MCC.

## INTRODUCTION

SARS-CoV-2, the causative agent of COVID-19, continues to circulate widely following its entry into the human population in 2019. In most cases, the first site of SARS-CoV-2 infection is the conducting airways^1–3^. These sections of the airway are lined with a pseudostratified epithelium blanketed in mucus, which is produced by goblet and other secretory cells. The mucus acts as a barrier between the environment and the epithelium, trapping particulate matter including pathogens. Multiciliated cells lining the airway move the mucus away from the respiratory airways and towards the pharynx, keeping the alveolar surface clear for gas exchange. This process of mucociliary clearance (MCC) is a key innate immune barrier; defects in MCC as seen in primary ciliary dyskinesia and cystic fibrosis are associated with increased frequency and severity of respiratory infections^4^.

While MCC can inhibit initial infection, its role in viral spread following establishment of an infection is less clear. If virions are shed from the apical surface of infected epithelial cells, ciliary beating and the movement of the mucus layer may facilitate spread. For example, Respiratory Syncytial Virus (RSV) has been found to form cometlike foci in model systems with functional MCC, suggesting its spread may be facilitated by mucociliary movement^5^. MCC has been considered in *in silico* modeling of early stages of intra-host SARS-CoV-2 and influenza A virus (IAV) spread, but little experimental data on the impact of MCC on these viruses is available^6,7^.

MCC is a complex, dynamic system, with feedback mechanisms between mucus composition, periciliary liquid composition, and ciliary motion patterns that ultimately determine the physicochemical properties of the mucus and its movement^8^. Viral infection has been shown to induce changes in MCC, both through the direct modulation of infected cell function as well as indirectly through induction of innate immune and inflammatory signaling pathways^9–11^. SARS-CoV-2 infection is known to increase mucus secretion, and it is thought that multiciliated cell function is impaired as infected cells become dysfunctional and die^10,12,13^. However, the details of how and when MCC changes during infection remain unclear.

Primary human bronchial epithelial cells (HBEs) grown at an air liquid interface (ALI) support MCC and are commonly used to model the airway in studies of infectious disease^14–22^. However, fixed timepoint imaging of these cultures has resulted in only limited understanding of MCC dynamics, particularly given the heterogeneity of ALI cultures. Here, we use live imaging of SARS-CoV-2 infected ALI cultures to track mucus secretion, ciliary motion, and viral spread^1,23^. We find that, similar to RSV, MCC appears to facilitate the spread of SARS-CoV-2 early in infection. Later in infection, infected multiciliated cells largely retain ciliary motion while increased mucus secretion leads to inhibition of MCC. These results suggest a complicated interplay between the host MCC system and respiratory virus infection.

## RESULTS

### Inverted air-liquid interface cultures support live imaging of SARS-CoV-2 infection and mucociliary clearance.

**Figure 1:**
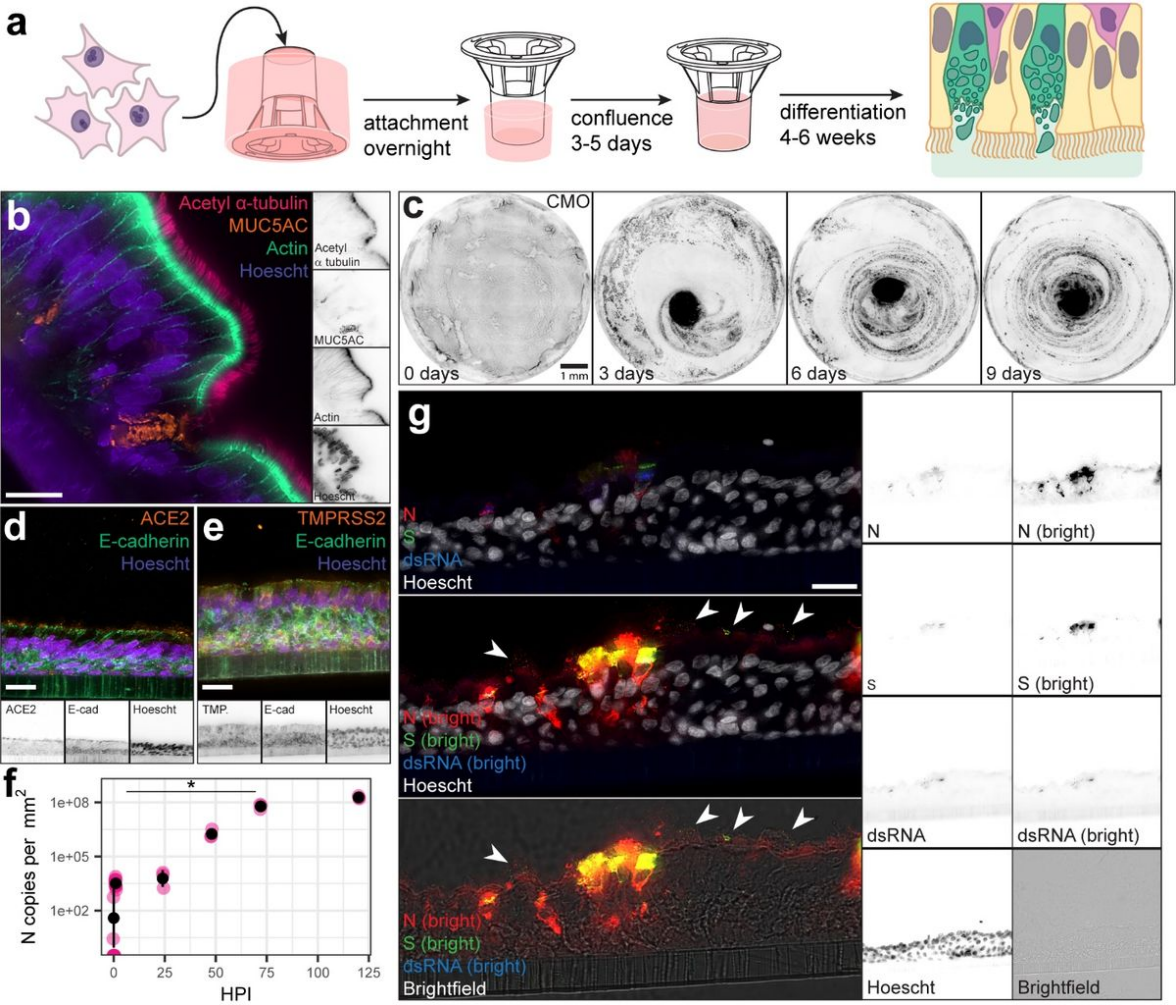
Inverted ALI culture models support SARS-CoV-2 infection and MCC. **a,**Schematic of inverted ALI culture. **b,** Immunofluorescent image of a section of a differentiated ALI culture stained for: cilia (acetyl α-tubulin, bright pink), cell-cell junctions (actin, spring green) mucus (MUC5AC, amber), and nuclei (Hoescht, electric indigo). Panels on the right show single channels in inverted grayscale. Scale bar = 20 µm. **c,** Time lapse of an inverted ALI culture incubated with CellMask Orange Plasma Membrane stain (CMO, inverted greyscale) demonstrating rotary mucociliary clearance over 9 days. Scale bar = 1 mm. **d,** Immunofluorescent image of a section of a differentiated ALI culture stained for ACE2 (amber), tight junctions (E-Cadherin, spring green), and nuclei (Hoescht, electric indigo). Bottom panels show single channels in inverted grayscale. Scale bar = 20 µm. **e,**Immunofluorescent image of a section of an ALI culture stained for TMPRSS2 (amber), tight junctions (E-Cadherin, spring green), and nuclei (Hoescht, electric indigo). Bottom panels show single channels in inverted grayscale. Scale bar = 20 µm. **f,** Copies of nucleoprotein (N) RNA per square millimeter of culture area in mucus collected from inverted ALI cultures immediately prior to or at several timepoints after infection. Fuchsia points represent the mean of technical duplicates (n >= 3 biological replicates per timepoint). Black points and bars represent the mean of means +/− standard deviation. * p-value = 0.02 by Welch’s t-test. **g,**Immunofluorescent images of a section of a differentiated ALI culture 72 hours after SARS-CoV-2 infection stained for SARS-CoV-2 N (red), Spike (S, green), double-stranded RNA (dsRNA, blue), and nuclei (Hoescht, white). Thresholding of viral antigens is altered in the middle & bottom panel to highlight dim viral protein puncta among the cilia and mucus (white arrowheads). Panels on the right show single channels in inverted grayscale. Scale bar = 20 µm.

To model the physiology of the human airway in a format amenable to live imaging, we used an inverted primary human bronchial epithelium air-liquid interface (ALI) culture system following the method of Zaderer et al. (Fig. 1a)^23^. Briefly, primary HBEs were seeded on the underside of permeable transwell supports to enable high-resolution live imaging of the apical surface of the cells through an inverted microscope. After differentiation, these cultures developed a characteristic pseudostratified morphology resembling human bronchial epithelium with multiciliated cells and goblet cells as confirmed by immunostaining for markers of cell-cell junctions (actin), cilia (acetyl a-tubulin), and mucus (MUC5AC) (Fig. 1b). While these largely formed a monolayer on the structured support, some ALI cultures contained furrows and intraepithelial lumens of varying connectivity henceforth referred to as crypts (Supp. Figure 1). Donors differ in their propensity to form these structures, as previously observed^24^.

To track the motion of mucus on the apical surface, we stained live cultures with either CellMask Orange Plasma Membrane (CMO) or NucView 530 (NV). CMO labels the plasma membrane upon application to the apical surface, but after 1–2 hours accumulates in dead cells and presumptive membrane debris in the mucus (Supp. Video 1). NV, a fluorogenic caspase 3/7 substrate, labels apoptotic cells, which are shed into the mucus allowing persistent monitoring of MCC (Supp. Video 2). As illustrated by CMO staining, the apical mucus disc in inverted ALI cultures often rotated over time, indicative of coordinated MCC (Fig. 1c, Supp. Video 2). Where rotary MCC was not present, the mucus flowed in a disorganized manner consistent with uncoordinated patches of locally coordinated multiciliated cells (Supp. Video 1)^25^. To observe multiciliated cells, we stained live cultures with SPY650-tubulin (Supp. Video 3). Imaging cultures with this dye revealed that the epithelium is sometimes quite dynamic, with cell migration leading to formation & dissolution of densely ciliated furrows (Supp. Video 4). The brighter structures visible in SPY650-tubulin correspond to the ciliated furrow and crypt structures visible in sections. 4/114 cultures had patchy ciliation with a hypermobile phenotype (Supp. Video 5). SPY650-tubulin also labels cilia with sufficient intensity to acquire high framerate videos of ciliary motion and determine ciliary beat frequency (Supp. Video 6). Taken together, these data confirm that the inverted ALI culture model resembles human bronchial epithelium and supports MCC for live imaging.

Immunostaining of inverted ALI cultures for ACE2 (the SARS-CoV-2 receptor) and TMPRSS2 (the major protease cofactor), confirmed that these cells express both factors required for SARS-CoV-2 entry at the apical surface of the epithelium and among the cilia (Fig. 1d, e). To assess the permissivity of these cultures to SARS-CoV-2 infection, we inoculated cultures with SARS-CoV-2 USA/WA1–2020 at a multiplicity of infection (MOI) of 1 via the apical surface, then quantified viral nucleocapsid (N) RNA in apical rinsates (mucus eluted in PBS) over time by quantitative PCR (qPCR) (Fig. 1f). To avoid disturbing the replication kinetics, each infected culture was only used for one rinsate timepoint. The concentration of N RNA in the mucus increased over time, consistent with productive viral replication and release from the apical surface. Maximum N concentration was observed at 120 hours post infection (HPI) at 1.9 × 10^8^ N copies per square millimeter of culture surface area.

To confirm permissivity, we infected differentiated ALI cultures with SARS-CoV-2 as above and stained for viral replication compartments with anti-double-stranded RNA (dsRNA) antibodies or for viral antigens with anti-N or anti-Spike (S) antibodies at 72 HPI (Fig. 1g). Each marker of infection was detectable above background and displayed distinct localization patterns within the epithelium. S primarily localized in both a perinuclear compartment as well as at the apical surface of the cells below the cilia. N localized more broadly within the cytoplasm with occasional staining in the cilia. dsRNA occurred in a perinuclear compartment, consistent with the formation of membrane-associated replication complexes. Puncta of co-localized S and N occur within the cilia & mucus layer (Fig. 1g, white arrowheads). Punctate protein antigen staining generally did not co-localize with dsRNA, suggesting that at least some of the puncta represent actual virions rather than autofluorescent or noninfectious debris. If virions are released into mucus, that suggests that MCC could be relevant for viral particle spread. Overall, these results indicate that inverted ALI cultures are a suitable model for studying early SARS-CoV-2 spread in the context of mucociliary clearance.

### Live imaging of inverted ALI cultures reveals kinetics and spatial aspects of SARS-CoV-2 spread.

**Figure 2:**
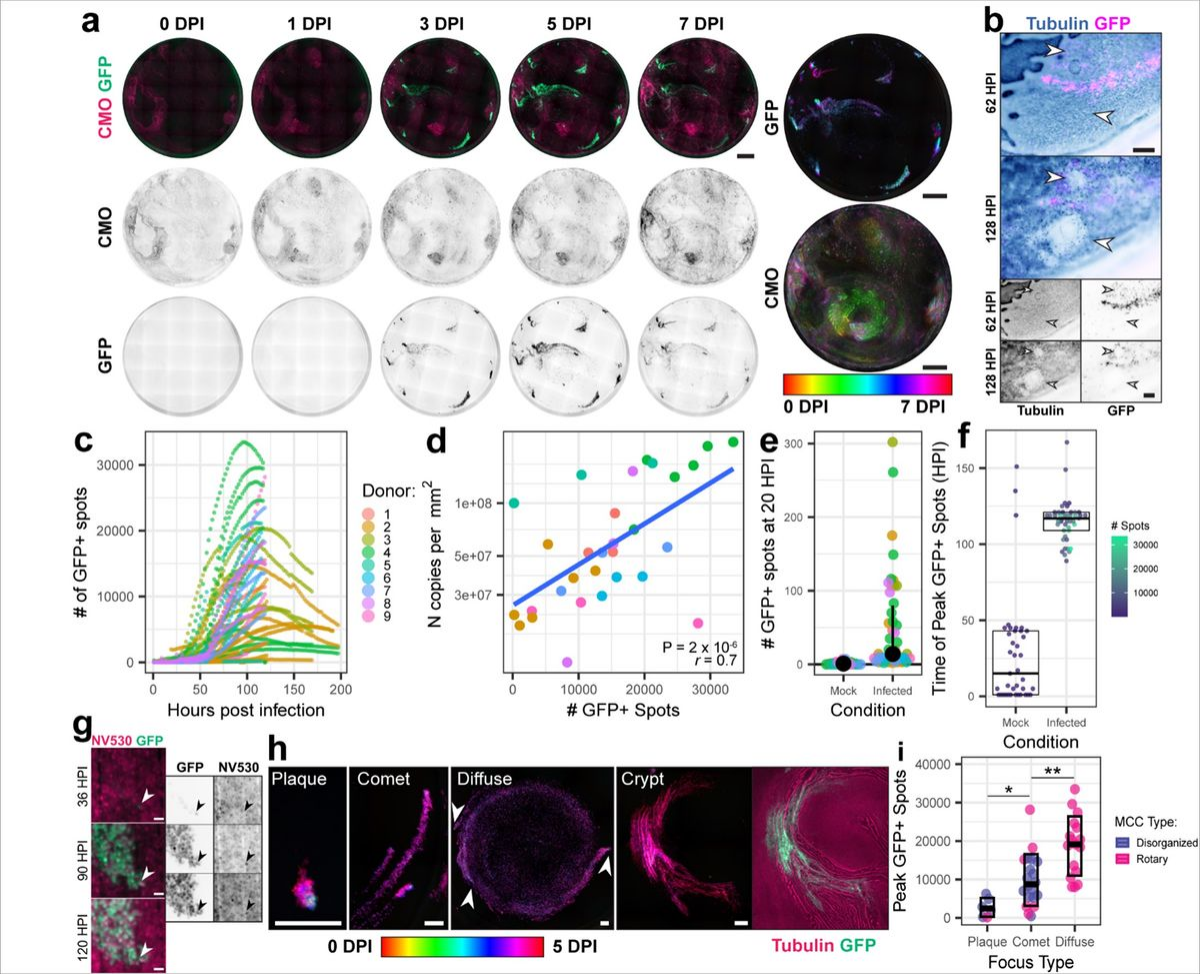
Spatiotemporal dynamics of SARS-CoV-2 spread in inverted ALI cultures. **a,** Differentiated ALI cultures were infected with icSARS-CoV-2-eGFP (green) in the presence of the indicated stains and imaged using a 4x air lens every other hour for 5–9 days. Images of one representative culture stained with CellMask Orange Plasma Membrane dye (CMO, bright pink) and infected with icSARS-CoV-2/eGFP (spring green) are shown over 7 days of infection. Temporal projections of this culture are shown on the right. Scale bar = 1 mm. **b,** Representative time lapse images of a focus of infection (GFP, magenta) in a culture stained with SPY650-tubulin (cyan ink wash). Arrowheads highlight ruptures. Scale bar = 200 µm. **c,** Graph of GFP+ spots over time in SARS-CoV-2 infected ALI cultures from 9 independent donors (n >= 3 replicates per donor). **d,** Scatterplot of peak GFP+ spots versus N RNA concentration per square millimeter of culture area at 5 days post infection (mean of technical duplicate) from infections of ALI cultures from 8 independent donors (n >= 3 replicates per donor). Pearson’s correlation coefficient r = 0.7, p-value = 2 × 10^-6^. **e,** The number of GFP+ spots counted manually at 20 hours post-infection for each culture (n >= 3 cultures per condition). The black dot and bar depict the mean and interquartile range. **c,d,e,** Colors are consistent for each donor. **f,** Time to peak GFP+ spots for cultures where peak occurred prior to cessation of imaging (n = 3–6 cultures per donor per condition). Dots are colored by peak number of GFP+ spots. Black boxplots depict the mean and interquartile range. **g,** Representative time lapse images of a comet-shaped focus of infection, with SARS-CoV-2-eGFP in spring green & NucView 530 in bright pink. The white arrowhead indicates the first observed infected cell. Scale bar = 10 µm. **h,** Representative images of foci of various morphology. Scale bar = 100 µm. The first four images are temporal projections from 0 to 5 days post infection; the image to the far right shows the crypt focus at a single timepoint with icSARS-CoV-2/eGFP in green and SPY650-tubulin in bright pink. Arrowheads mark comet heads which initiated the diffuse focus. **h,** Peak GFP+ spots in each culture separated by focus morphology type. Points are colored by MCC pattern of the culture. Mean and standard deviation are shown. * p = 0.004 & ** p = 0.0006 by Wilcoxon rank sum test.

To characterize SARS-CoV-2 spread within the bronchial epithelium, we performed live imaging of inverted ALI cultures after infection with a SARS-CoV-2 USA/WA1–2020 derived fluorescent reporter virus that has eGFP inserted into the ORF7a locus (icSARS-CoV-2/eGFP)^1^. Differentiated ALI cultures from 9 independent donors were infected in technical triplicate and imaged longitudinally for up to 9 days post-infection. Using CMO to simultaneously track MCC, we observed that most cultures had comet-like foci of infection that closely followed the tracks of the mucosal discs (Fig. 2a, Supp. Video 7). When visualized by temporal projection (Fig. 2a, right panel), the motion of the mucus (yellow and green) was observed to precede the development of infection foci (cyan). Similar results were obtained when using NV to track MCC and SPY650-tubulin to track ciliary motion (Supp. Video 8, Supp. Figure 2). The epithelium often appeared to rupture and heal in infected cultures, with 18/23 surveyed infected cultures having ruptures. (Fig. 2b, Supp. Video 9). Ruptures occurred both within foci and among bystander cells. No ruptures were observed in untreated uninfected cultures (0/19 surveyed).

To quantify infection kinetics, we measured the number of GFP + spots at each frame, subtracting spots present at baseline (generally attributable to background around the rim of the transwell) (Fig. 2c). The peak number of GFP + spots and the peak GFP + fractional area were positively correlated with the number of copies of N RNA in apical rinsate at 120 HPI, validating the metric (Fig. 2d). Isolated GFP + cells first became apparent around 20 HPI, with a median of 14 initial infected cells per culture (Fig. 2e). The viral inoculum contained 5 × 10^5^ plaque-forming units (PFU, titered on Vero E6 cells) per well, corresponding to an MOI between 0.5 and 1, so the relative scarcity of early infected cells suggests there are formidable barriers to initial infection in these cultures. After the initial stage of infection, there was considerable heterogeneity in the infection kinetics both within and between donors. For most cultures, the number of GFP + spots increased rapidly around 48–72 HPI, peaking at a median of 103 HPI (Fig. 2f). Infected cells generally maintained viability and persisted within the epithelium for multiple days (Fig. 2g, Supp. Video 10). Nevertheless, overall infection remained relatively low, with peak infection in the most infected culture only ever reaching 3.3 × 10^4^ GFP + spots or 20% of the total surface area. While the density of GFP + cells could be much higher in infected foci, large swathes of the surface remained uninfected in most infected cultures.

Notably, infection kinetics in different cultures correlated with the morphology of the infection foci and with MCC patterns (Fig. 2h, i). In some cultures, infection was restricted to small, circular plaque-like foci with a high density of infected cells in small, defined areas. These cultures generally had disorganized MCC and a low peak infection rate. Most cultures had comet-like foci, with a single infected cell initially appearing around 16–20 HPI and multiple infected cells appearing subsequently downstream of MCC tracks around 36–48 HPI. These cultures generally had larger areas of coordinated MCC, though it could be more disorganized or more rotary. A third subset of cultures had infection appear diffusely over the entire surface of the culture around 36–48 HPI. These cultures reached the highest peak infection and all displayed efficient MCC in a rotary pattern (Fig. 2i). Note that a fourth focus type was occasionally observed that appeared restricted to subepithelial crypts or furrows in the culture. These foci were identified by the presence of horizontally oriented cells in patterns matching the whorls of the cilia marker, which becomes quite bright in the crevices and crypts of the epithelium. Such foci grew rapidly but were restricted to the crypt to the extent that the crypt had limited access to the broader apical surface. Taken together, these data suggest that MCC can impact the spatiotemporal dynamics of SARS-CoV-2 spread.

### MCC facilitates SARS-CoV-2 spread.

**Figure 3:**
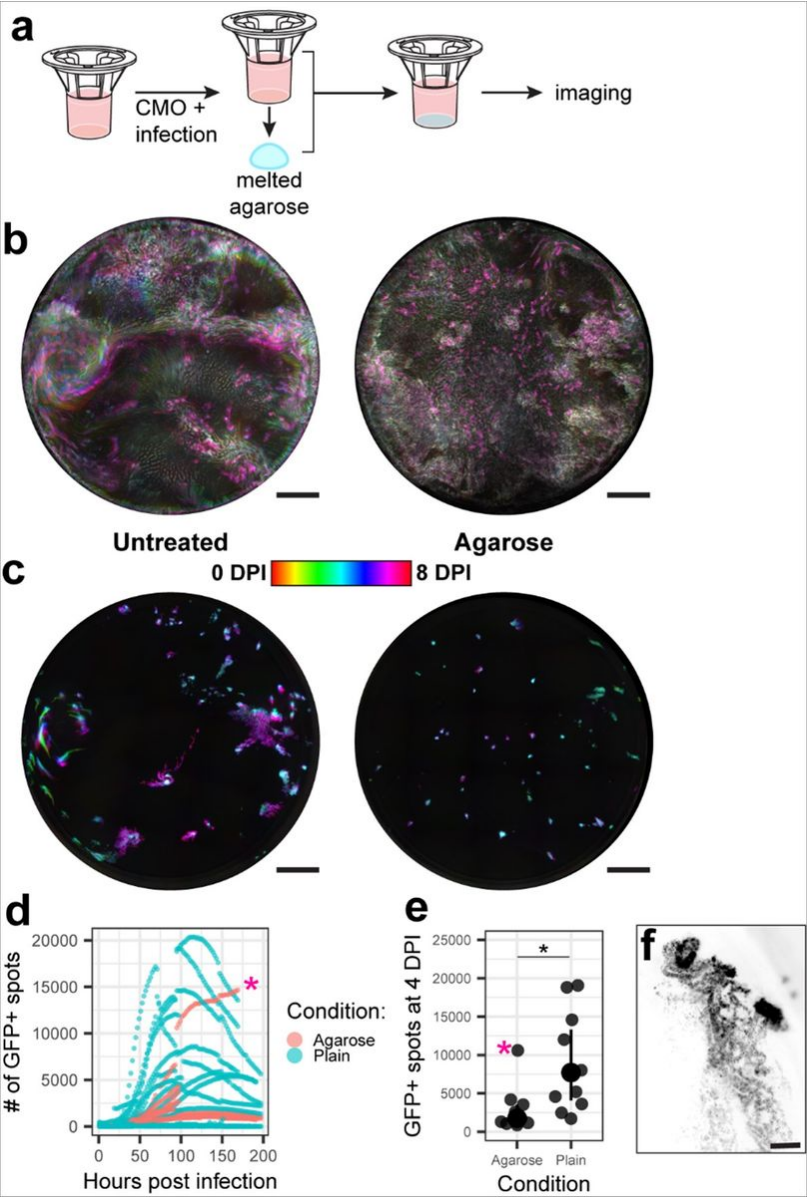
Agarose overlay restricts mucus flow and SARS-CoV-2 spread. **a**, Schematic of agarose overlay experimental design. **b**, Temporal projection of CMO signal and **c,** SARS-CoV-2-eGFP signal in representative cultures with (right) and without (left) agarose overlay. Scale bar = 1 mm. **d**, The number of GFP+ spots in each culture at each timepoint post infection. Agarose overlay cultures are in red, and no overlay cultures are in blue (n >= 3 replicates per donor per condition for 3 donors). The pink asterisk marks an outlier with infected crypts. **e**, The number of GFP+ spots at 4 days post infection for each culture shown in **d**. The pink asterisk marks the same outlier. * p = 0.007 by Wilcoxon rank sum test. **f**, Inverted greyscale image of the GFP channel at 3 days post infection of an infected crypt in the agarose overlay culture indicated with a pink asterisk in **d** & **e**. Scale bar = 100 µm.

Since MCC patterns in individual cultures correlated with the morphology of SARS-CoV-2 foci, we hypothesized that restriction of MCC would limit SARS-CoV-2 spread. To mechanically restrict MCC, we applied a layer of low melting temperature agarose to the apical surface of the cultures immediately after mucus removal, CMO staining, and infection (Fig. 3a, Supp. Video 11). As seen in the temporal projection of the CMO channel (Fig. 3b), overlaying the cultures with agarose restricted mucus movement as reflected in the larger areas of static gray (right) as opposed to more saturated colors showing movement over time (right). Imaging of icSARS-CoV-2/eGFP spread following agarose overlay in multiple donors showed a clear restriction of infection to small, plaque-like foci (Fig. 3c, Supp. Video 11) similar to those observed in individual cultures with disorganized MCC above (Fig. 2g, h). Quantification of this effect in multiple donors confirmed lower peak infection percentage and slower spread upon agarose overlay (Fig. 3d, e), except for one culture which supported infection in an intraepithelial crypt (Fig. 3f, Supp. Video 12). These crypts are not exposed to the apical surface and thus not accessible to the agarose overlay. Rapid spread within the crypt further suggests that agarose overlay specifically inhibits infection by MCC restriction as opposed to a nonspecific mechanism, such as hypoxia, osmotic changes, or thermal damage to cells during overlay application.

**Figure 4:**
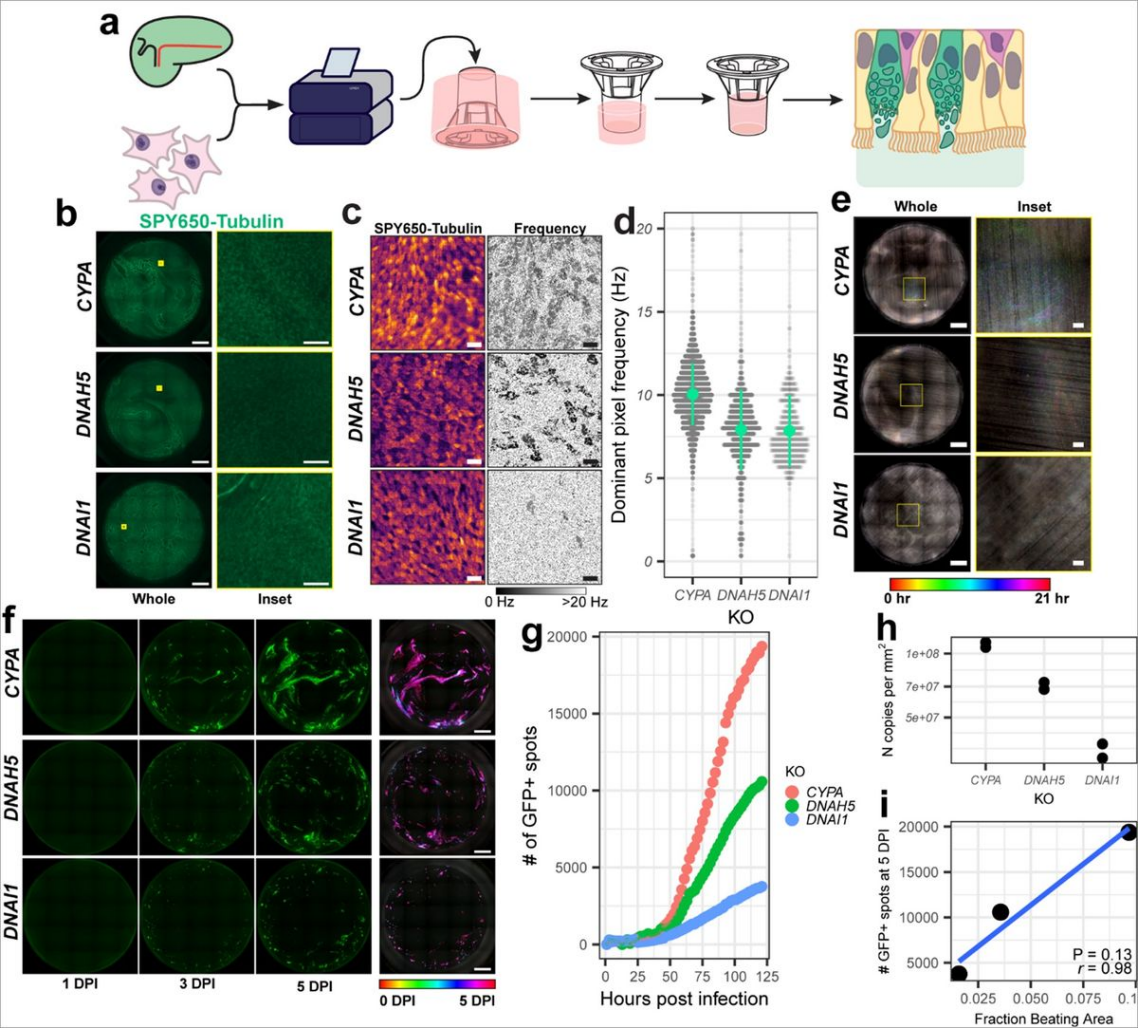
Genetic perturbation of ciliary motion inhibits SARS-CoV-2 spread. **a**, Schematic for the production of gene knockout in inverted ALI cultures. **b**, Images of SPY650-tubulin signal (green) from KO cultures. Left panels are whole cultures (scale bar = 1 mm). Right panels are insets marked with yellow in the whole culture images (scale bar = 50 µm). **c**, Sum projections of SPY650-tubulin videos in knockout ALI cultures (left). The dominant beat frequency in Hz of each pixel of the same movies (right) with beating multiciliated cells visible as dark grey patches. Scale bar = 20 µm. **d**, Bee-swarm plots of the distribution of beat frequency of pixels with power above the noise threshold in SPY650-tubulin movies of knockout ALI cultures. 0.3 mm^2^ per culture (~1% of total culture area) was surveyed per culture with differences in plot density reflecting different numbers of beating pixels in each culture. **e**, Temporal projections of 20-hour time lapses of NucView 530 signal from each knockout ALI culture during infection. Rainbow traces indicate motion, while whiter portions indicate lack of motion. Left panels are whole cultures (scale bar = 1 mm). Right panels are insets marked with yellow in the whole culture images (scale bar = 50 µm). **f**, Snapshots of GFP signal following SARS-CoV-2/eGFP infection of each knockout culture at the indicated time points. The rightmost panels are temporal projections of the entire time course. Scale bar = 1 mm. **g**, Number of GFP+ spots at each timepoint for each culture. **h**, Copies of nucleoprotein RNA per mm^2^ of culture area in mucus collected from knockout ALI cultures at 120 HPI. Points are technical duplicates. **i,** Scatterplot of peak GFP+ spots versus fraction beating area for KO cultures. Pearson’s correlation coefficient r = 0.98, p-value = 0.13.

To address this question in a different way, we knocked out axonemal dynein genes in primary HBEs prior to differentiation of inverted ALI cultures. Axonemal dyneins drive ciliary motion by generating the sliding motion of microtubule doublets within cilia^26^. *DNAH5* and *DNAI1* were targeted here on the basis of their common occurrence in human primary ciliary dyskinesia and the successful production of *DNAH5* and *DNAI1* KO mice, suggesting that these mutations are well tolerated^27,28^. After initial expansion, undifferentiated HBEs were electroporated with *in vitro* synthesized CRISPR-Cas9 ribonucleoproteins (crRNPs) targeting *DNAH5*, *DNAI1*, or *CYPA* as a control. Cells were seeded at high density on the undersides of collagen coated transwells (Fig. 4a). Multiciliated cells still differentiated and the apical surface of the cultures were ciliated as usual (Fig. 4b).

To determine the impact of these perturbations on ciliary motion, cultures were stained with SPY650-tubulin and visualized by high-speed video microscopy. Beat frequency was determined using a Fast Fourier Transform (FFT)-based power spectral density analysis. For each pixel, the power of the signal at each possible frequency (given the sampling rate, i.e. the framerate of the video) was calculated, and the frequency with maximal power was taken to be the dominant beat frequency (Fig. 4c)^29^. Most pixels with power greater than 40 corresponded to beating cilia, consistent with the SPY650-tubulin staining and the characteristic patchy appearance of the ciliary beat frequency images. Compared to the *CYPA* KO cultures, the *DNAH5* and *DNAI1* KO cultures had showed substantially diminished beat frequencies (Fig. 4c, d). Given the polyclonal nature of the KO cells, motile cilia were still evident in both axonemal dynein KO cultures, with the *DNAH5* KO culture having an intermediate phenotype and the *DNAI1* KO culture having very few motile cilia (Fig. 4c, d). MCC in each culture was additionally assessed by imaging with NV, which likewise showed movement of apoptotic cell debris in the apical space of the *CYPA* KO culture, but not in that of the axonemal dynein KOs (Fig. 4e).

Each culture was subsequently challenged with SARS-CoV-2/eGFP and imaged over 120 hours. The *CYPA* KO culture had comet-like foci that spread over time (Fig. 4f, Supp. Video 13). In contrast, infection of the *DNAI1* KO culture resulted in small plaque-like foci, similar to the agarose overlay, while the *DNAH5* KO culture had an intermediate phenotype consistent with its intermediate effect on ciliary motion (Fig. 4f, Supp. Video 13). This correlated with peak number of infected GFP + cells and levels of SARS-CoV-2 RNA in the apical rinsate (*i.e.*, the mucus) of each culture (Fig. 4g, h). In sum, these data suggest that mechanical and/or genetic perturbation of ciliary motion and MCC inhibits SARS-CoV-2 spread after infection.

### SARS-CoV-2 infection only modestly inhibits ciliary motion.

**Figure 5:**
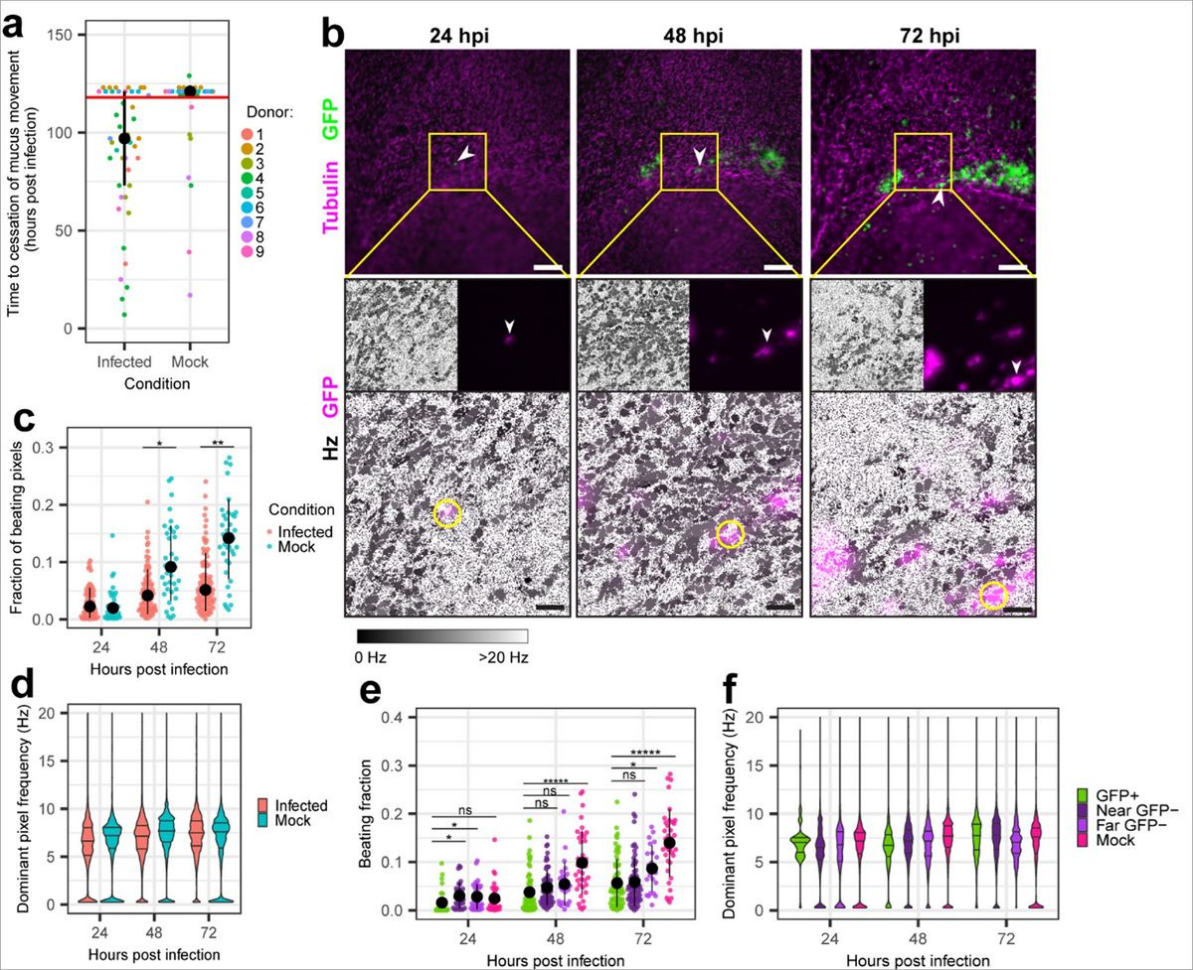
Effects of SARS-CoV-2 infection on ciliary motion. **a,** Time to cessation of mucus motion in each infected or mock culture (n = 9 donors, >= three replicates per donor). Black dots and lines represent the median +/− interquartile range of each condition. Points above the pink line had mucus motion continuing to the end of imaging. p = 3 × 10^−5^ by chi-squared test on the depicted data binned by whether MCC ceased during imaging. **b,** Live imaging snapshots of an infected cell identified at 24 hpi & followed to 72 hpi. Tubulin and SARS-CoV-2/eGFP are visualized in magenta and green, respectively (top). Scale bar = 200 µm. Beat frequency in Hz of the central field of view (FOV) is shown below in greyscale with infected cells overlaid in magenta (bottom). Scale bar = 50 µm. Arrowheads (top) or yellow circles (bottom) identify the initial infected cell. **c,** The fraction of beating pixels (power above 40) in each FOV for mock-infected (blue) and SARS-CoV-2 infected (red) cultures over time. Each FOV is 0.0274 mm^2 with at least eight FOVs surveyed for three donors at each timepoint. The black dot and line represents the average +/− standard deviation in each condition. Significance by Wilcoxon rank sum test. * p = 3 × 10^-5^. ** p = 7 × 10^-8^. **d,** Violin plots of beat frequency of each beating pixel for all fields of view shown in **c**. Beat frequency is cut off at 20 Hz to highlight the physiological range of ciliary motion. Horizontal lines within each violin mark median and interquartile range. **e,** The fraction of beating pixels in subsets of FOVs from mock and infected cultures stratified by proximity to GFP signal. Near GFP-pixels are from FOVs that contain GFP+ pixels and far GFP- pixels are from FOVs in infected cultures that do not contain any GFP+ pixels. Significance was determined by Kruskal-Wallis rank sum test with Benjamini-Hochberg post hoc correction at alpha = 0.05. * p 0.025–0.0025; ** p 0.0025–0.00025, ***** p < 0.0001 **f**, Violin plots of beat frequency of beating pixels in mock and infected cultures, with those from infected cultures stratified by proximity to GFP+ pixels. **d,f,** Beat frequency is cut off at 20 Hz to highlight the physiological range of ciliary motion. Horizontal lines within each violin mark median and interquartile range.

MCC facilitates the spread of SARS-CoV-2 early in infection, but this effect is limited. Comet-shaped foci do not grow indefinitely, suggesting that either downstream cells develop resistance to infection or that MCC becomes dysfunctional at later time points. In uninfected cultures, mucus movement most often continues for the duration of any given experiment (Fig. 5a). However, in SARS-CoV-2 infected cultures, mucosal movement typically stops at an average of 100 HPI (Fig. 5a). Previous studies have suggested that MCC dysfunction during SARS-CoV-2 infection may be driven by the loss of multiciliated cells, which are the major target of infection in airway epithelium, or by the enhanced secretion of MUC5AC-containing mucus by goblet cells^10,12^.

To differentiate between these possibilities, we first monitored ciliary motion of both infected and bystander cells in inverted ALI cultures from three donors at 24, 48, and 72 HPI. Cultures were stained with SPY650-tubulin to track ciliary motion. Five infected cells were identified at 24 HPI, when GFP signal first became detectable, and tracked over the next 48 hours (Fig. 5b). All five cells tracked over this time period continued beating at a regular beat frequency. A cell that is beating and ensconced within the monolayer must be alive and performing metabolic activities to support ciliary motion, so these findings suggest that multiciliated cells retain viability and function for days following SARS-CoV-2 infection. Furthermore, two of the five cells initiated foci during tracking, indicating that productive infection does not necessarily inhibit ciliary motion. Likewise, of the newly observed GFP + cells at 48 HPI, most continued to beat at 72 HPI.

To better survey the population beat frequency of the infected & bystander cells over time, we performed per pixel quantification of the above infections again using an FFT-based power spectral density analysis. The fraction of pixels with maximum signal power greater than 40 was used to approximate the culture area covered with beating cilia. The fraction of beating pixels in a field of view (FOV) increased over time in both mock and infected cultures, likely as a result of improved signal detection as the SPY650-tubulin incubation period increased (Fig. 5c). Between mock and infected cultures, the fraction of beating pixels was comparable at 24 HPI, but differed at 48 and 72 HPI with infected cultures showing fewer beating cilia. Despite the decrease in beating cilia coverage, many FOVs in infected cultures were dense with beating cilia and the beat frequency distribution of the cilia was qualitatively similar between mock and infected cultures (Fig. 5d), consistent with our cell-level observations described above. The shape of the ciliary beat frequency distributions was bimodal, with one population of pixels beating around 7 Hz and another approaching 0 Hz. The higher frequency population reflects beating cilia while the lower frequency population appeared to arise from pixels that contain debris moving through the field of view. This suggests that while individual multiciliated cells may retain viability and functionality after infection, there are fewer beating cilia in infected cultures over time.

To understand whether these differences between mock and infected cultures are driven by infected cells or bystander cells, we compared the fraction of beating area and beat frequency between neighboring GFP + and GFP- pixels in the same fields of view (infected & near bystanders), GFP- pixels in fields of view that contained no GFP + pixels in infected cultures (distant bystanders), and all pixels from mock cultures (Fig. 5e, f). The fraction of beating cilia (*i.e.*, pixels with a power > 40) significantly differed between neighboring GFP + and GFP- pixels only at 24 hpi, and differences between the groups within infected cultures were dwarfed by the overall mock vs. infected difference (Fig. 5e). Furthermore, the beat frequency distribution was qualitatively similar between neighboring GFP+ & GFP- pixels (Fig. 5f). One caveat to these analyses is that motile cilia tend to present as two patches of pixels with a defined beat frequency to either side of the cell body, corresponding to the start and end positions of the ciliary beat. As such, the frequency value and the signal intensity of a given pixel may not necessarily correspond to the same cell, potentially diluting differences between infected cells and their neighbors. Nevertheless, these data suggest that decreases in ciliary motion during infection are due to impacts on both infected and bystander cells, rather than specifically infected cells.

**Figure 6:**
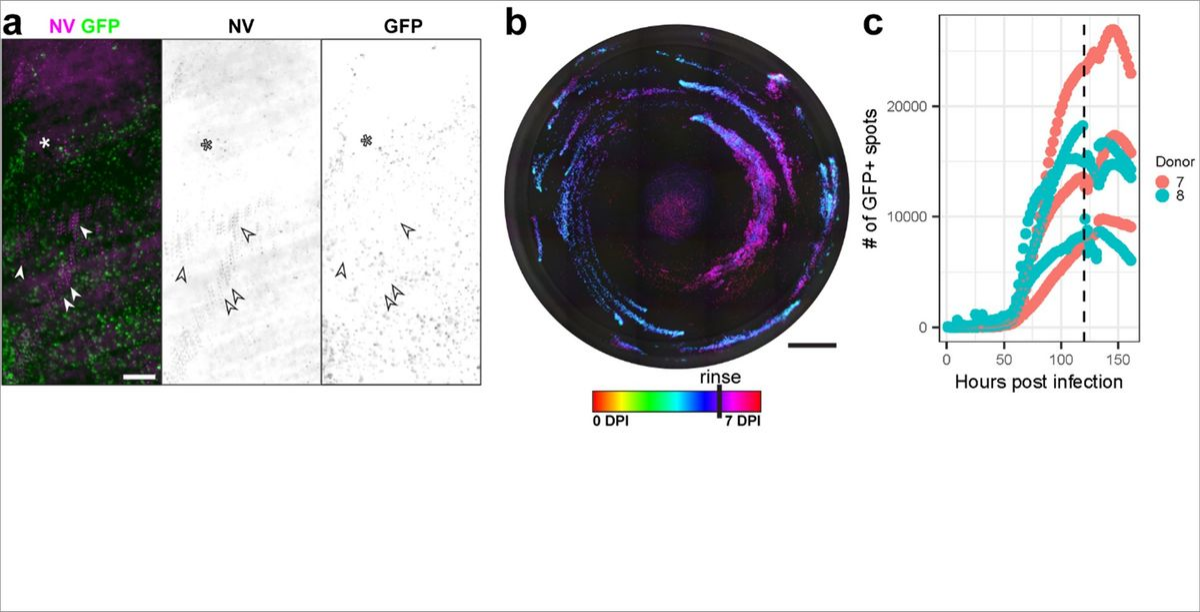
Effects of SARS-CoV-2 infection on mucociliary clearance. **a,** Projected image showing apoptotic nuclei tracks (NucView 530, magenta) moving over a focus of infection (GFP, green) over the course of 0.8 seconds. Bottom panels are inverted greyscale single channels. Arrowheads point to moving NV puncta; asterisk is adjacent to static NV puncta. Scale bar = 200 µm. **b,** Temporal projection of the GFP channel of a whole infected culture with a mucosal rinse at 120 hpi (one representative image). Scale bar = 1 mm. **c,** GFP+ spots at each timepoint for 120 hpi rinsed cultures (n = 3 replicates for two donors). The vertical dashed line marks the time of rinse.

Besides ciliary motion, the other major variable contributing to MCC is mucus secretion. To test whether the mucus influenced the spatial restriction of infection at later timepoints, we rinsed cultures at 120 HPI, shortly after the peak of infection. After rinsing, we observed apoptotic cells moving in mucus over foci of infected cells, consistent with active MCC (Fig. 6a). Interestingly, in some fields of view apoptotic cells appeared to move more quickly over infected foci than over adjacent bystanders (Fig. 6a, asterisks & arrowheads). Cultures that displayed rotary MCC early in infection resumed rotary MCC after rinsing, and those with comet-like foci saw the extension of the comets (Fig. 6b, Supp. Videos 5 & 14). The number of GFP + spots also increased after rinse in each culture (Fig. 6c). This suggests that stalled infection and cessation of MCC in infected cultures may be due to secretion of a soluble factor, likely mucus, and not solely depletion of infected multiciliated cells. Taken together, these data support a complicated role for MCC in SARS-CoV-2 infection, acting as an innate barrier for initial infection while serving to facilitate spread after infection is established.

## DISCUSSION

Here we demonstrate methods to perform live imaging of SARS-CoV-2 infected ALI cultures, revealing some of the complexities of how MCC impacts viral infection. Though MCC is an efficient barrier to initial penetration, after infection MCC facilitates a large burst size by spreading virions caught in mucus. Later in infection, altered mucus secretion inhibits MCC and restricts growth of foci of infection. The paradoxical effects of MCC in early infection spread have been partially appreciated previously, with some observations of vectorial spread in RSV and human parainfluenza virus, and some models of dissemination within the airway incorporation assumptions about vectorial spread^5,7,30^. Our live imaging reveals that this vectorial spread enables large numbers of cells to be infected essentially at once, after the initial infected cell begins to release progeny virions, leading to a large burst of infection and more rapid infection kinetics.

Though MCC-mediated spread seems plausible for any respiratory virus with apical entry and shedding, viruses may differ in the extent to which they develop the characteristic cometlike foci in ALI culture. Measles virus develops dense plaques, the en bloc shedding of which may be key to measles’ exceptional transmissibility^31,32^. RSV and human parainfluenza virus may display comet shaped foci early in infection, but for these viruses the foci merge and grow to occupy the majority of the culture surface area, rather than remaining distinct as in SARS-CoV-2 in the current study^5,30^. For influenza A virus, while comet shaped foci have been anecdotally reported (personal communication), it appears to be a relatively uncommon phenomenon and no published images were identified^33,34^. Comparisons between viruses based on published literature are suspect owing to differences in infection, culture conditions, and imaging protocols. For example, even in SARS-CoV-2 where we see cometlike foci with frequency, published en face images are often plaquelike^17,35^. With that caveat, differential interaction with components of MCC may represent a novel facet of host-pathogen interaction. Viruses may modulate their focus phenotype & spread kinetics by altering their cytotoxicity, interactions with mucus, or cytokine secretion. For example, influenza viruses modulate their interactions with mucus & the glycocalyx with their surface hemagglutinin and neuraminidase activities^36,37^. Balance between the two activities affects mucus penetrance and could lead to different focus morphologies between strains with different activity levels.

The extent to which alterations in MCC early in infection may be adaptive for the host or the pathogen is unclear. For example, it has been reported that influenza A virus infection can upregulate ciliary motion and MCC very early in infection^9^. Is this a strategy for the virus to produce an early large burst, or a strategy for the host to avoid further infectious insults once compromised? For a virus like SARS-CoV-2, which is sensitive to interferons but induces them only late in infection, hijacking MCC to produce an early large burst of infectious particles in the airway may be a strategy to infect the next host before antiviral signaling begins.

Later in infection, it is well established that SARS-CoV-2 impairs MCC^10,12^. Reports on human disease find increased mucus secretion and damaged ciliated epithelium^38^. In ALI cultures increased MUC5AC secretion has been observed, and the finding that SARS-CoV-2 preferentially infects multiciliated cells has led some to conclude that multiciliated cell depletion is a major cause of MCC dysfunction in SARS-CoV-2 infection^10,12^. Our work complements these studies by showing that while there is an increase in dysfunctional ciliated cells, the impact of that dysfunction is relatively minor and mucus secretion is likely the major factor limiting MCC in infection.

Live imaging is a particularly useful tool for ALI culture studies of host-pathogen interactions. ALI cultures are the gold standard for cell culture-based studies of the human airway, expressing similar profiles of surface proteins & proteases to native epithelium, but vast within-donor heterogeneity and the challenges of scaling up ALI culture can make answering questions about dynamic & variable host-pathogen interactions unfeasible. The set of tools we have developed and applied allows us to characterize in detail and follow the course of infection spatially within individual cultures, permitting inferences that would be challenging to make based on fixed samples. The combination of SPY650-tubulin and NV is particularly useful since it allows observation without perturbing the apical surface, and because apoptotic cells ejected from the epithelium are a native substrate of MCC, obviating concerns about bead material and chemical properties. This presents an improvement over existing methods for labeling cilia and mucus flows, which rely on dyes or beads applied to the apical surface either in large volumes of diluent or by manual pipetting of small volumes directly onto the culture surface. The use of air lenses with a high depth of field also facilitates long term imaging by preserving the air liquid interface. No specialized or excessively costly tools are required, so this method should be readily transferrable to other groups. There are still substantial limitations to the system, notably that in most humans mucus is unidirectionally transported out of the body and does not accumulate and require manual removal, as is the case in transwell-based ALI cultures.

Other limitations of live imaging of viral infection include the inherent tradeoffs of a reporter virus. We use a reporter virus lacking ORF7a^1^. SARS-CoV-2 ORF7a is known to have various roles in infected cells including BST2 and MHC class I antagonism^21,39^. While its effects on apoptosis and cell viability have not been characterized, SARS-CoV-1 ORF7a is a homolog with known conserved interactions, and it is known to induce apoptosis^40–42^. We find that infected cells retain viability and ciliary function for days after infection. This could be a mechanism by which the virus facilitates its spread- an infected cell that can remain functional as it produces virions, moving them along to encounter more potential host cells, may spread infection farther than a cell that dies more quickly or loses the ability to spread its progeny by MCC. It is possible that most wild type strains of SARS-CoV-2 would kill infected cells more quickly. However, there are a variety of mutations found in ORF7 in circulating SARS-CoV-2 strains that could affect its function during infection^43,44,45^.

People with conditions that impair MCC are generally considered to be more vulnerable to respiratory infections. However, this vulnerability is largely to bacterial pathogens, and when increased risk of adverse COVID-19 outcomes is observed, it is typically in subpopulations with advanced lower lung disease secondary to bacterial colonization^46,47^. In this study MCC impairment inhibits viral replication, similar to what has been observed in human parainfluenza virus, which produces restricted plaques in ALI cultures made with cells from donors with cystic fibrosis^30^. Since viral infection likely spreads to the lower lung by inhalation of aerosols generated during speech and breathing, it is possible that the smaller plaques and lower viral load we see are still sufficient to seed the lower lung, where the pathology occurs^1^.

## METHODS

### Inverted ALI culture.

Primary bronchial epithelial cells (Lonza CC-2540) were initially expanded to 20–50 million cells in PneumaCult Ex Plus medium (Stemcell #05040) on collagen coated flasks, then cryopreserved in aliquots of 7.5 × 10^5^ cells. To produce inverted ALI cultures, aliquots from this initial passage were further expanded in collagen coated 75 cm^2^ flasks, then trypsinized and seeded on the underside of 0.4 µm pore polyester cell culture supports (Corning #3470 or Celltreat #230635). Briefly, prior to seeding, the undersides of the inserts were coated with 0.1 mg/mL type I collagen (Advanced Biomatrix #5005), equilibrated to 37°C, and then placed upside down in 12 well plates flooded with warm Ex Plus medium sufficient to maintain moisture on the polyester membrane. 10^5^ cells in 100 µL media were gently pipetted onto the exposed underside of the membrane and returned to the incubator. The 12 well plates were fitted with 3D printed adaptors to raise the lids to avoid contact with the cell suspension. After overnight attachment, cell culture supports were inverted and placed in 24 well plates with Ex Plus medium in the basal and apical compartments. Upon confluence, cultures were fed with PneumaCult ALI medium (Stemcell #05001) in the cup of the cell culture support (Fig. 1a) and an air liquid interface was established by removing media from the 24 well plate. ALI medium was changed daily for the first few days, then every 48–72 hours thereafter. Mucus was removed by apical PBS (Corning 21–040-CV) rinse weekly from the 3rd week or the beginning of mucus secretion. To rinse, cultures were incubated with 600 µL PBS at 37°C and 5% CO2 for 30–45 minutes, then the loosened mucus and excess PBS were aspirated. Cultures were considered differentiated when mucus secretion and ciliary motion were evident, from 4–6 weeks. Experiments were performed with 6–12 week old cultures. During infetions media changes were performed as usual, but mucus rinses were deferred for infections lasting longer than one week unless specifically stated otherwise.

### Cells & viruses.

Vero E6 and Vero CCL81 cells were maintained at 37°C and 5% CO2 in DMEM (Corning) supplemented with 10% fetal bovine serum (Atlanta Biologics), 1x PSG (Gibco), 10 µM HEPES (Gibco), 1 µM sodium pyruvate (Gibco), and 1x MEM NEAA (Sigma). SARS-CoV-2 USA/WA1–2020 (BEI NR-52281) and ic-SARS-CoV-2-eGFP (BEI NR-54002) were propagated in Vero CCL-81 cells (ATCC CCL-81). Virus stocks were thawed, then diluted in PBS with 3% BSA, 1.1 mM CaCl2, and 2.2 mM MgCl2. 90% confluent flasks were incubated with 0.01 MOI virus for 1 hour at room temperature, rocking every 15 minutes. Serum free media (as described above sans FBS) was added to the flask. Cells were incubated at 5% CO2 and 37°C for 48 hours, then supernatant was harvested, centrifuged at 4000 x g for 10 minutes to reduce cell debris, aliquoted, and stored at −80°C. An uninfected flask was processed in parallel to produce an equivalent supernatant for mock infections. Virus was titrated by plaque assay. All experiments involving replication competent SARS-CoV-2 were performed in a Biosafety Level 3 facility following approved standard operating procedures at Northwestern University or at the Sanford Burnham Prebys Medical Discovery Institute.

### Viral quantification by plaque assay.

Viral stocks were serially diluted in DMEM, then incubated on confluent Vero E6 cells at warm room temperature (28°C) for 1 hour, rocking every 15 minutes. The inoculum was then removed, the monolayer rinsed twice with PBS, and the cells covered with 1.2% microcrystalline cellulose (Sigma 435244) MEM. 72 hours after infection, the carboxymethylcellulose overlay was carefully removed and the monolayers fixed and stained with 4% formaldehyde, 20% ethanol, and 0.2% crystal violet. Plaques were manually counted.

### Viral quantification by qPCR.

Apical rinsates from infected ALI cultures were inactivated by heating to 60°C for 30 minutes. RNA was extracted using a QIAmp Viral RNA Mini Kit (cat #52906) per manufacturer’s directions. Viral load determination was performed by quantitative reverse transcription and polymerase chain reaction (qRT-PCR), using the CDC 2019-nCoV RT-PCR Diagnostic Panel with N1 and RNase P probes as previously described [https://www.cdc.gov/coronavirus/2019-ncov/lab/rt-pcr-panel-primer-probes.html]. Specimens were excluded based on their cycle thresholds (Ct) for RNase P, with those above 35 Ct being excluded due to insufficient quality for further analysis. N copy number was calculated based on linear regression of a standard curve with known N quantities.

### Generation of KO primary bronchial epithelial cells.

Cas9 ribonucleoprotein complexes were complexed in vitro as previously described^48^. Briefly, lyophilized tracrRNA & crRNA (Dharmacon) were resuspended to 160 uM, combined, and incubated at 37°C for 30 minutes to form an RNA duplex at 80 uM. An equal volume of 40 uM Cas9 protein solution (UCB Macrolab) was added and incubated at 37°C for 15 minutes to produce crRNPs at 20 uM concentration. Target sequences: DNAH5, AGACCCGAGAAAGATCTCGT; DNAI1, GATGAATACCGGGACCAGGT; CYPA, AGGTCCCAAAGACAGCAGGT. Early passage HBEs were then trypsinized at 50–75% confluency, counted, separated into aliquots of 100,000 cells per reaction, and spun down at 200 x g x 5 minutes. Single reaction aliquots were then resuspended in 20 µL Lonza nucleofection buffer P3, mixed with 3.5 µL crRNP solution, placed in wells of a 16-well cuvette, and electroporated using program EH100 on a Lonza 4D X unit. Immediately after nucleofection, cells were mixed with 80 µL prewarmed Pneumacult Ex Plus medium and returned to the incubator for 30 minutes. Cells were then moved to collagen coated 12 well plates with prewarmed Pneumacult Ex Plus medium and allowed to recover for approximately two days, when the 12 well plates approached 70% confluency. At this point cells were seeded on the underside of transwell membranes as described above.

### Infection of ALI cultures.

Mucus was removed from the apical surface of the culture by incubating with PBS for 30–45 minutes at 37°C, then carefully aspirating residual mucus and PBS. 100 µL viral inoculum diluted in PBS to contain 5 × 10^5^ PFU (0.5 MOI) was placed in the bottom of a 24 well plate (Fisherbrand or Greiner) and ALI cultures placed on top such that the apical surface contacted the inoculum droplet. Cultures were then incubated 1 hour at warm room temperature (28°C) with intermittent rocking. The apical surface was then rinsed twice with PBS and cultures incubated at 37°C with 5% CO2. To quantify viral load in mucus, infected cultures were rinsed with PBS as prior to infection; residual mucus and PBS was carefully pipetted off and pooled with the remainder of the rinsate. Rinsate was stored at −80°C until assayed by qPCR or plaque assay. For mucus qPCR, mucus samples were inactivated by heating to 60°C for 30 minutes prior to analysis outside the BSL3. For immunofluorescence, cultures were inactivated and fixed by overnight incubation at 4°C in 4% formaldehyde in PIPES pH 6.8 buffer.

### Agarose overlay.

Prior to infection, low melting temperature agarose (Sigma) was set melting on a heat block. After removal of the inoculum and PBS rinse as described above in the general infection protocol, residual PBS was thoroughly aspirated from the apical surface to ensure the agarose would have adequate contact with the culture to restrict mucus motion. 30 µL of melted agarose was spotted onto a clean surface and just prior to solidification, the freshly dried and infected transwell was stamped into the spot to produce a thin layer of agarose over the apical surface.

### Live imaging of ALI cultures.

Live imaging was performed on a DeltaVision Elite microscope outfitted with stagetop incubator. Premixed blood gas was routed through an aquarium bubbler column filled with distilled water kept in the stagetop incubator to humidify and warm it to 37°C, then piped onto cultures at 20 mL/min. Cultures were imaged in a glass bottomed 24 well plate (Cellvis) with outer wells and interstitial spaces filled with distilled water to further maintain humidity and provide additional thermal mass. A custom 3D printed adaptor was used to fit the CO_2_ frame lid to the 24 well plate. To mitigate condensation, the imaging cassette was equilibrated on the stage for at least 15 minutes prior to imaging and after any excursion to the hood. For movies of entire cultures, imaging was done in a 5×5 panel of 1024 × 1024 fields of view across 4 Z planes spanning 80 um with a 4x air lens (Olympus UPLXAPO4X), with panels acquired every 2 hours. For ciliary motion movies, 256×256 pixel fields of view were acquired with a 10x air lens at 125 frames per second. Centered on the smaller field of view of each ciliary motion video, 1024 × 1024 reference images of all channels spanning approximately 20 microns of Z were also acquired with the same 10x lens. Prior to imaging, cultures were stained with various dyes. Cellmask Orange Plasma membrane (Invitrogen C10045) was applied to the apical surface diluted 1:1000 in PBS for 7 minutes at 37°C, then rinsed thrice with PBS. SPY650-tubulin (Spirochrome SC503) was mixed in the media at 1:2000 48–72 hours prior to imaging and maintained in media throughout. NucView 530 was mixed in the media at 1:500 shortly before initiation of imaging and maintained in media throughout.

### Image processing: whole culture movies.

Initial processing was performed in ImageJ. Panels were stitched using the Grid/Collection stitching plugin^49^. Stitched images were concatenated to assemble full time series, Z projected, then registered using the Linear Stack Alignment with SIFT Multichannel plugin to align across timepoints and media changes. GFP + spots were quantified using Trackmate 7, using a Laplacian of Gaussian spot detector^50^. Images were visualized using various lookup tables, including ‘Cyan Ink Wash’ (https://sites.imagej.net/JDM_LUTs/).

### Ciliary beat frequency measurement.

256×256 pixel videos of SPY650-tubulin cultures were acquired at an 8 ms framerate for 3 seconds using a 10x air lens. For each pixel, an FFT based method was used to assign a dominant frequency and a power associated with that frequency. Beat frequency images were then matched back to the corresponding position in Z-projected multichannel reference images. Code was written in Python & is available on github (https://github.com/markebecker/ali_imaging/).

### Immunofluorescence.

Uninfected cultures were fixed in 4% formaldehyde in pH 6.8 PIPES buffer at 4°C overnight, then excised from the plastic transwell support and embedded in OCT. After cryosectioning onto superfrost slides, 10–12 um sections were blocked with normal donkey serum and 0.5% Triton X for 45 minutes, then stained with primaries diluted in equal parts phosphate buffered saline and normal donkey serum. Primary incubations were all for 1 hour at 37°C. Anti acetyl alpha tubulin (Cell Signaling Technology D20G3) was used at 1:800. Anti MUC5AC (Abcam 45M1) and anti S (Sino 40150-D003) were used at 1:200. Anti dsRNA was used at 1:1000 and anti N at 1:500 (Sino 40143-T62). Donkey derived fluorescent secondaries (Jackson ImmunoResearch) with appropriate species specificity were diluted 1:500 in PBS and incubated on sections for 25 minutes at room temperature alongside Alexa Fluor^™^ 488 Phalloidin (Invitrogen A12379). Slides were stained with Hoescht 33432 then mounted with Dako wet mount and sealed with nail polish. To mitigate section loss, staining was performed with an open source derivative of the Sequenza coverslip staining platform. Slides were imaged on a DeltaVision Ultra or Elite widefield scope.

## Supplementary Material

Supplement 1
