# Supplementary material for: Live imaging of the airway epithelium reveals that mucociliary clearance modulates SARS-CoV-2 spread
Source: Res Sq. 2023 Sep 8:rs.3.rs-3246773. Preprint. [Version 1] doi: 10.21203/rs.3.rs-3246773/v1 (PMC10503848; doi:10.21203/rs.3.rs-3246773/v1)
Supplement: Supplement 1 [file NIHPPRS3246773V1-supplement-1.pdf]

Supplementary Files

This is a list of supplementary files associated with this preprint. Click to download.

- SV1.mp4
- SV2.mp4
- SV3.mp4
- SV4.mp4
- SV5.mp4
- SV6.mp4
- SV7.mp4
- SV8.mp4

- SV9.mp4
- SV10.mp4
- SV11.mp4
- SV12.mp4
- SV13.mp4
- SV14.mp4
- SupplementaryFigures.docx
